# Investigation of High Voltage Polymeric Insulators Performance under Wet Pollution

**DOI:** 10.3390/polym14061236

**Published:** 2022-03-18

**Authors:** Ali Ahmed Salem, Kwan Yiew Lau, Zulkurnain Abdul-Malek, Wenbin Zhou, Salem Al-Ameri, Samir A. Al-Gailani, Rahisham Abd Rahman

**Affiliations:** 1 Institute of High Voltage and High Current, School of Electrical Engineering, Universiti Teknologi Malaysia, Johor Bahru 81310, Malaysia; ahmedali.a@utm.my (A.A.S.); zulkurnain@utm.my (Z.A.-M.); 2 Department of Mechanical Engineering, Imperial College London, London SW7 2AZ, UK; 3 Faculty of Electrical and Electronic Engineering, University Tun Hussein Onn Malaysia, Batu Pahat 86400, Malaysia; rahisham@uthm.edu.my; 4 School of Electrical and Electronic Engineering, Universiti Sains Malaysia, Nibong Tebal 14300, Malaysia; samer.algailani@usm.my

**Keywords:** polymer, polluted insulators, conductance, FEM, electric field, current density

## Abstract

In this paper, a unique approach based on electrical characteristics observed from measurements of contaminated polymeric insulators was established to calculate the electric field distribution over their surfaces. A case study using two different 33 kV polymeric insulator geometric profiles was performed to highlight the benefits of the proposed modeling approach. The conductance of the pollution layer was tested to establish a nonlinear field-dependent conductivity for pollution modeling. The leakage current (LC) of the polluted insulator was measured in a laboratory under clean and wet conditions. Then, using the finite element method (FEM), the electric field and current density distributions along the insulator were computed. The results showed that the insulators experienced an increase in the electric field (EF) magnitude ranging from 0.3 kV/cm to 3.6 kV/cm for the insulator with similar sheds (type I) and 2.2–4.5 kV/cm for the insulator with alternating sheds (big and small, type II) under the high rain condition with a flow rate of 9 L/h. Meanwhile, the highest electric field under fog was 1.74 kV/cm for the insulator with similar sheds and 2.32 kV/cm for an insulator with alternating sheds. Due to the larger diameter on the big shed and the longer leakage distance on the insulator with alternating sheds, the EF on the insulator with alternating sheds is higher than the EF on the insulator with similar sheds. The proposed modeling and simulation provided a detailed field condition estimation around the insulators. This is critical for forecasting the emergence of dry bands and the commencement of flashover on the surfaces of the insulators.

## 1. Introduction

Polymeric insulators are a vital part of electrical power transmission and distribution networks, with a single insulator failure potentially causing the entire power system to collapse catastrophically. The insulators are constantly subjected to environmental contamination, such as industrial wastes, chemicals, and natural pollutants. The contamination led by the sea wind is deposited in the form of salt on the surface of the insulator in coastal locations. Meanwhile, under industrialized environments, pollution is deposited in the form of dust and ashes [1,2,3]. Once the pollutants over the surface of the insulator absorb moisture, a conductive layer forms, allowing the leakage current (LC) to flow on the insulator surface. Water vapours evaporate as a result of resistive heating due to system voltages, resulting in the formation of dry bands (dry regions on the insulator surface). Electric forces will be combined with the working voltage throughout the dry bands, resulting in cracking and degradation in the insulator surface [4,5,6]. Under exaggerated conditions, arcs are created, extending over numerous dry bands, causing a full insulator breakdown and power interruption.

Electric power companies increasingly use polymeric insulators for overhead transmission and distribution lines. However, their long-term efficiency and dependability are unknown because they have a shorter operational lifetime than traditional glass and porcelain insulators [7]. To verify the performance of polymeric insulators, significant research has been conducted using theoretical and experimental methodologies [8,9,10,11]. Many of them are concerned with calculating the electric field surrounding the insulators. The study of the electric field provides an insight into pollution concerns, such as aging and quick deterioration. Moreover, dry band formation prediction may be undertaken with greater accuracy [12].

It is difficult to measure the voltage and distribution of electric field around realistic insulators, and it is significantly more difficult when the surface is contaminated. The electrostatic probe [13,14,15] approach is prone to mistakes. However, they may be reduced by employing sophisticated and advanced electric field sensing devices. Therefore, numerical techniques are used to examine insulators’ surrounding electric fields. Computational approaches, such as the finite element method (FEM) [16,17], boundary element method (BEM) [18], and the charge simulation approach, can be used [19]. Compared to experimental work, which necessitates a complex setting and a lengthy testing time, the computing approaches are more cost-effective. Furthermore, modern numerical tools can solve complicated field models faster and precisely.

In the literature, when modeling outdoor insulators, researchers commonly assume that the pollution layer has a single and linear conductivity [20,21,22,23,24]. In an actual scenario, this presumption may not always be appropriate. The tangential component of the electric field significantly impacts surface conductivity. The conductivity reduces with time when humidity evaporates from the contaminated layer due to surface heating [25,26,27,28,29]. The pollution layer’s drying impact will account for the electric field strength. Experimental findings of low voltage layer conductance testing were used to derive the suggested nonlinear electrical attribute.

The current study presents the experimental investigation of the current and conductance of polluted insulators based on the electric field under various applied voltages. In addition, the modeling approach for the distribution of electric fields and current density on insulator surfaces was performed using the FEM technique. Under a clean and wet environment, a three-dimensional structure is adequately modeled, but due to the symmetry of the insulator, the insulator was effectively described by a two-dimensional model due to axisymmetry. In the testing of insulators, fog and rain were taken into account. The insulator profile was also considered. Hence, two polymer insulators with different architectures were used in this investigation. The FEM simulation of the pollution scenario, useful in forecasting dry band development, was applied.

## 2. Experimental Work and Method

### 2.1. Insulators Structure

**Figure 1:**
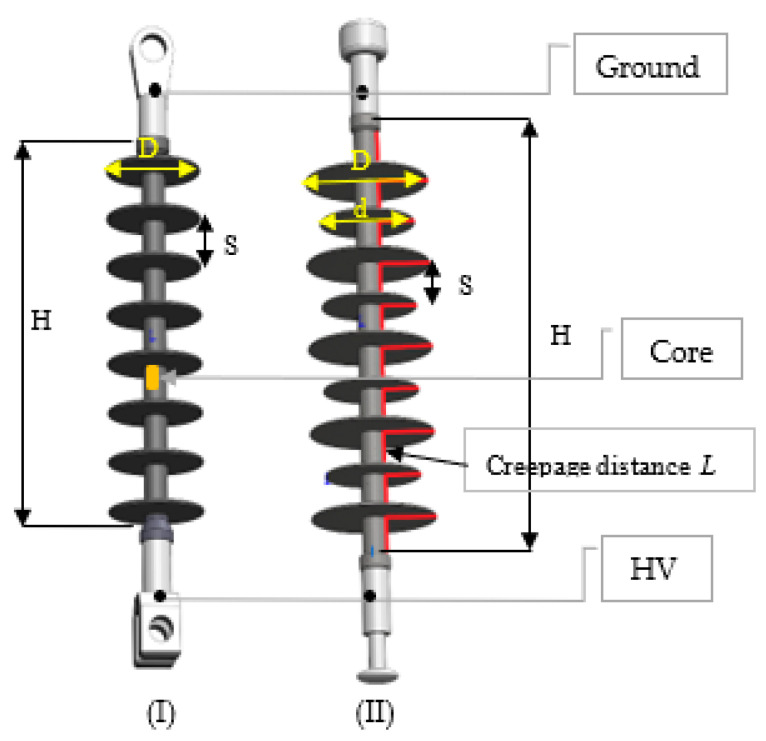
Geometries of type (**I**) and type (**II**) insulators, where insulator type (**I**) has a constant shed diameter *D* while insulator type (**II**) has alternating big and small sheds with diameters *D* and *d*, respectively.

The geometries of the test insulators are illustrated in Figure 1. As illustrated in Figure 1, the test samples employed in this study are two different types of 33 kV polymeric insulators, named as type I and type II. The two insulator designs were utilized in the study to explore insulator conductance and current. Insulator type I has a constant shed diameter *D*, whereas insulator type II has alternating big and small sheds with diameters *D* and *d*, respectively. Table 1 shows the essential electrical characteristics of the test specimens, in which *D* and *d* are the large and small shed diameters, respectively, *S* is the distance between the sheds, *L* represents the creepage distance, *H* is the insulator height, and *L/H* is the creepage factor.

### 2.2. Laboratory Setup and Test Producers

**Figure 2:**
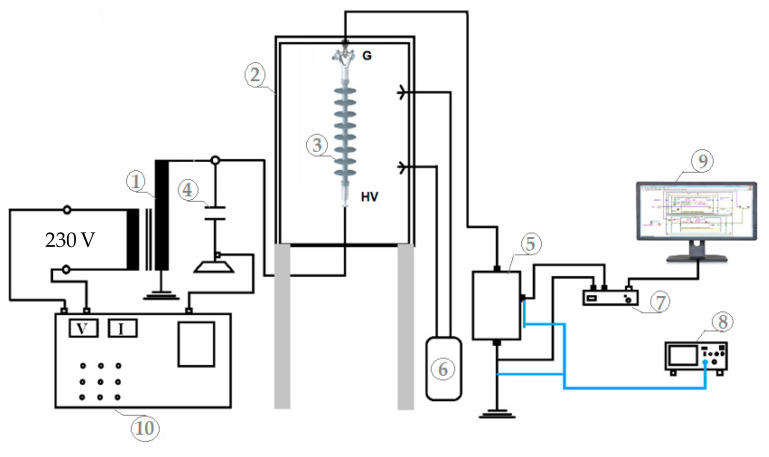
Experimental test setup consisting of (1) Transformer; (2)Test chamber; (3)Insulator sample; (4) Capacitive divider; (5) Shunt resistor; (6) Fog generator; (7) Data acquisition system (DAQ); (8) Oscilloscope; (9) Computer; (10) Control panel.

The leakage current and layer conductance tests were carried out by utilizing a fog chamber test equipment. The laboratory test equipment includes a single-phase AC 0.230/300-kV, 150-kVA, 50-Hz transformer that generates a voltage of up to 300 kV to energize the tested insulators, controlled from the control panel. The humidity in the test chamber was controlled using a fog generator. The flashover voltage of the insulator was measured using a capacitive divider. Simultaneously, a shunt resistor was used to measure the leakage current. The insulators were placed in the test chamber with walls made of 50 × 50 × 75 cm polycarbonate sheets. The data acquisition system (DAQ) was used to monitor the leakage current data. The data were sent from the DAQ to a personal computer (PC), where they were stored as a comma-separated values (CSV) file and presented on a graphical user interface in LabVIEW software. The oscilloscope was also used to validate the data from the DAQ. The insulator experimental circuit is shown in Figure 2. Figure 2 shows the connection of the test equipment. The tests were carried out under low voltage in accordance with the methods reported in the IEC 60,507 standard [30]. The insulators were subjected to various levels of low voltages, namely, 500 V, 1 kV, 1.5 kV, 2 kV, and 2.5 kV, for about 1 h at each voltage level. The wetting process began simultaneously as the electricity was turned on. In the meantime, the leakage current and conductance were measured. The wetting process ended when the pollutant layer achieved its maximum conductance value or when the pollution layer became saturated with water, with the leakage current measurement maintained.

### 2.3. Pollution Preparation

In this work, a solid layer technique was utilized to pollute the test sample [31,32]. The contaminated solution was made by combining 40 g of kaolin in 1000 mg of distilled water. To achieve the desired conductivity at 20 °C, sodium chloride (NaCl) was added in accordance with the IEC 60,507 standard criteria for severe pollution. Before applying contamination, the test insulators were carefully cleaned with detergent and water. The contaminated layer was wetted on the surface of the insulator using the ‘flow on’ approach. The polluted samples insulators were then left at room temperature for more than 7 h to dry before testing. If pollution was not dispersed consistently throughout the sample surface, the insulators were re-contaminated until the required level of uniformity was achieved. A contaminated layer from the sheds was carefully removed and dissolved in 1000 mg distilled water to test the electrical conductivity. It is worth noting that the pollution layer in this test consists of two types of pollutants: (a) soluble pollutant, measured with equivalent salt deposit density (ESDD), such as salt NaCl; (b) non-soluble pollutant, measured with non-soluble deposit density (NSDD), such as kaolin, sand, etc. The electrical conductivity of the solution was then measured with the Senso-Direct SN conductivity meter. Next, the salinity and the equivalent salt deposit density (ESDD) was determined as [32]:

(1) $$S_{a}={\left(5.7\times \sigma_{20}\right)}^{1.03}$$

(2) $$ESDD=\left(S_{a}\times V\right)/A$$

where *S_a_*, *σ_20,_ A*, and *V* are pollution solution salinity in mg/cm^3^*,* pollution layer conductivity at 20 °C in mS/cm, insulator surface area in cm^2^, and solution volume in cm^3^, respectively. Moreover, 5.7 is the constant of variation of the power function for the relationship between salinity and conductivity at 20 °C [30]. Following the measurement of the ESDD, the polluted water was filtered out, and the filter was dried and weighed. The non-soluble deposit density (NSDD) was computed as follows:

(3) $$NSDD=10^{3}(w_{f}-w_{i})/A$$

where *w_f_* is the weight of contaminated filter paper, *w_i_* is the weight of dry filter paper, and *A* is the area of the insulator surface.

Before beginning the low voltage test, a leakage current measurement was done to ensure the consistent pollution level on each test insulator. The insulator was applied with a relatively modest voltage (2 kV), which was sufficient to create a quantifiable leakage current. It was only used briefly to minimize surface heating and evaporation. The measured conductance was used to determine the degree of insulator pollution within a 10% standard deviation error. If the standard deviation error on the conductance value exceeded an acceptable range, the insulator was cleaned and contaminated again to achieve the desired conductance level. The high standard deviation error value indicated that the samples were not contaminated using the correct method or that there was an error in the test procedure.

The conductance of the contaminated layer was calculated using the leakage current readings from this experiment:

(4) $$G=\frac{I}{V_{C}}F$$

where *I*, *Vc*, and *F* are the current, the critical voltage, and the form factor of the insulator, respectively. Equation (5) is used to calculate the form factor *F* based on the insulator structure [30]:

(5) $$F=\int_{0}^{L}\frac{1}{2\pi r\left(l\right)}dl$$

where 2πr(l) is the insulator circumference at partial creepage distance *l*. *L* is the insulator creepage distance.

### 2.4. Wetting Process

This study considers two climatic conditions: fog and rain. The artificial wetting activity in the pollution test simulates the natural wetting process.

#### 2.4.1. Fog

Tiny water droplets flowed sluggishly and randomly in a foggy wetting case with a rate of 1.5 L/h. The wetness practically reached all insulator surfaces in any direction. Consequently, the foggy wetting process on the insulator surface was assumed to be uniform. It is worth mentioning that the contamination layer is deemed wetted and saturated once it achieves the maximum value of surface conductivity. Accordingly, this contamination layer will take time to moisturize.

#### 2.4.2. Rain

The characteristics that control the development of dry bands are dissipated power and wetting rate (the rate of moisture deposited on the surfaces of the insulators). Dry bands occur when the drying rate is equal to or greater than the wetting rate. Dry bands and surface discharges are often less serious when faced with severe rain. Rain might wash away contaminants and re-wet the dry areas on the insulator surface, lowering the likelihood of electrical discharges. However, when filthy insulators are exposed to drizzle or light rain, problems occur.

Rain, unlike fog particles, falls at a variable rate. When the rate of rain increases, so does the wetness of exposed surfaces. The flow rate is determined by the amount of water that reaches the insulator surface per hour. In this investigation, the contaminated layer was examined at three different flow rates: 9 L/h (high), 6 L/h (medium), and 3 L/h (low). Regardless of the angle of rain flow, wetting is considered uniform throughout the whole insulator surface in this situation. Therefore, the contaminated insulator was evenly wetted using the spray method. Using the suggested wetting method, water distribution on the surface of the insulator may be easily regulated without worrying about the washing effect or the time required to achieve optimal conductivity.

## 3. Experimental Results

### 3.1. Surface Conductance Assessment

**Figure 3:**
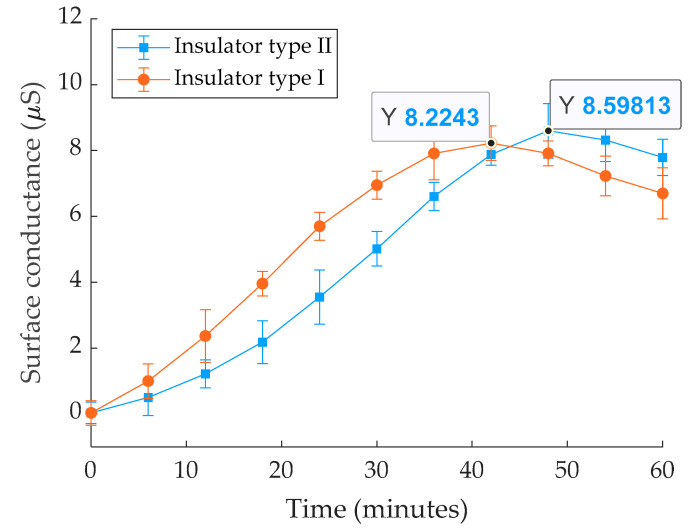
Leakage conductance behavior for insulator type I and insulator type II with respect to time, under fog and ESDD = 0.15 mg/cm^2^.

**Figure 4:**
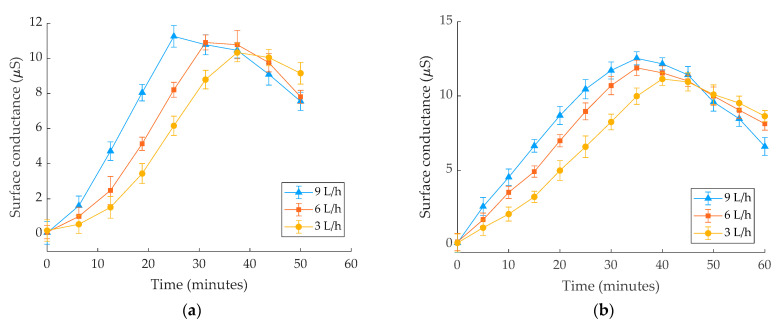
Surface conductance behavior of (**a**) insulator type I; (**b**) insulator type II under different rain flow rates.

Figure 3 shows the experimental outcomes of leakage conductance measurements on the pollution layer of insulators (ESDD = 0.15 mg/cm^2^) for 50 min. It can be seen that the relative error bar for all tests was less than 5%, indicating that the testing and polluting methods used were satisfactory. According to these results, the highest conductance conduction occurs when the insulators get saturated with water. After being wet with water for more than 0.5 h, the pollution layer is exposed to a high moisture content, imposing a maximum layer conductance. The maximum conductance value for insulator type I was about 8.22 μS at 43.2 min and 8.59 μS for insulator type II after 48.1 min. The maximum insulators values of conductance for five tests, average conductance, and standard division were listed in Table 2. The insulator structure affects the speed of wetting, and the conductance layer reached the maximum value faster in insulator type I. Figure 4 depicts the conductance fluctuation in the wet pollution layer (ESDD 0.15 mg/cm^2^) during the test for water flow rates of 3 L/h, 6 L/h, and 9 L/h for both insulators. For example, maximum conductance values in the insulator type I ranged between 10.2 and 11.22 µS for flow rates ranging from 3 to 9 L/h. The graph indicates that the variation in conductance is related to the water flow rates. Higher flow rates expedite wetting, and the conductivity layer reaches its maximum value faster.

### 3.2. Leakage Current Results

**Figure 5:**
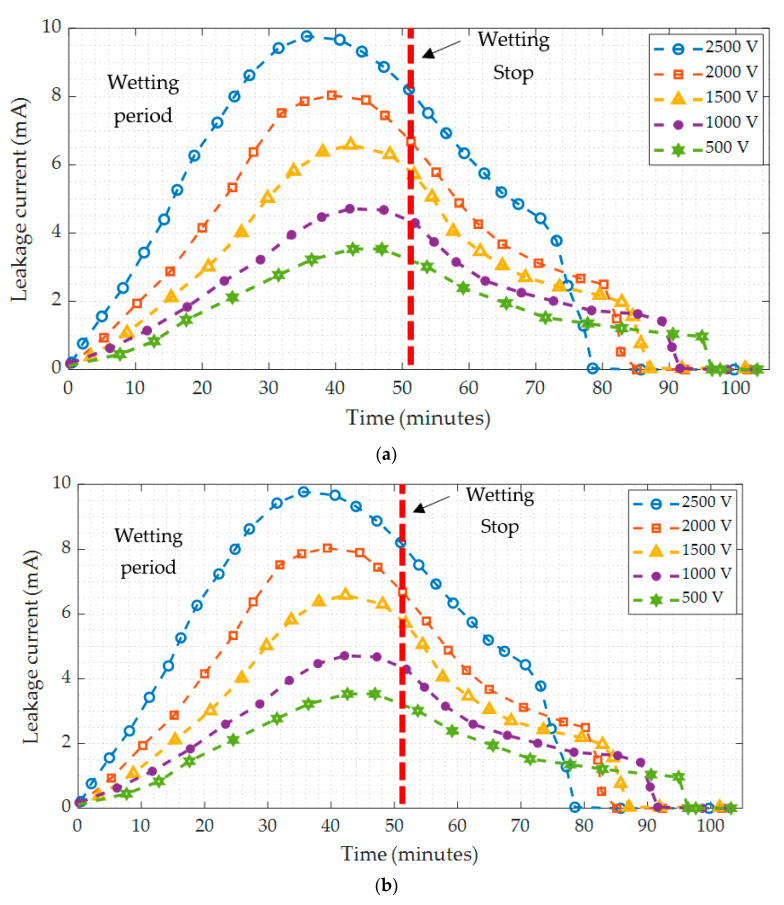
Leakage current of (**a**) insulator type I; (**b**) insulator type II under various applied voltages.

The leakage current was recorded throughout the test, and the time-varying leakage current curves are presented as in Figure 5. The leakage current test was performed under five voltage levels, with ESDD of 0.15 mg/cm^2^ and fog wetting for 52 min. According to Figure 5, the energizing voltage has a considerable impact on increasing the leakage current during the pollutant wetting process on the insulator surface. With increasing applied voltage, the time taken for the current to reach maximum point will decrease. Using insulator type I as an example, the duration from the start of the wetting process to the moment when the leakage currents are maximum is 53.2, 52.1, 39.6, 36.4, and 33.2 min when the applied voltage is 500 V, 1 kV, 1.5 kV, 2 kV, and 2.5 kV, respectively. The reason for this is the rapid response of the leakage current for the applied voltage under the wetting process of the pollution layer.

The current flowing through the contamination layer steadily increases as wetting continues. However, when wetting is stopped, the current decreases over time, as shown in Figure 5. This is due to surface evaporation caused by Joule heating during the voltage’s applied period. The sharp gradient of the leakage current after wetting stops, due to energizing, indicates an accelerated evaporate state that aids in drying the wet contaminated layer. The value of leakage current falls when the moisture degree in the contaminated layer lowers, and there is not enough heat energy to affect a further drop. As a result, minor fluctuations in leakage current are noticed over time. Because of the creation of dry bands on the surface of the insulator, the surface conductivity rapidly decreases near the end. Variations in the applied voltage observe the time required for this fast decline. Higher voltages, which provide a higher temperature as predicted, take less time to generate a dry band, whereas lower voltages require a longer period, as seen in Figure 5. When the intermittent dry bands occur, there is a series of abrupt fluctuations in the leakage current, especially at high levels of the voltage (1500 V, 2000 V, and 2500 V), indicating the existence of intermittent conduction.

The leakage current values on different insulators under the same conditions are significantly diverse. However, the following are some of the causes:

(1) The pollution layer’s surface conductance is related to the insulator’s geometrical form factor as in Equation (10).
(2) The wetting time of the pollutant layer on various types of insulators varies. The quickest to soak the pollutant layer is the type I insulator. In contrast, the wetting of the pollutant layer on the type II insulator takes longer. The causes are also connected to the construction of the insulators.

**Figure 6:**
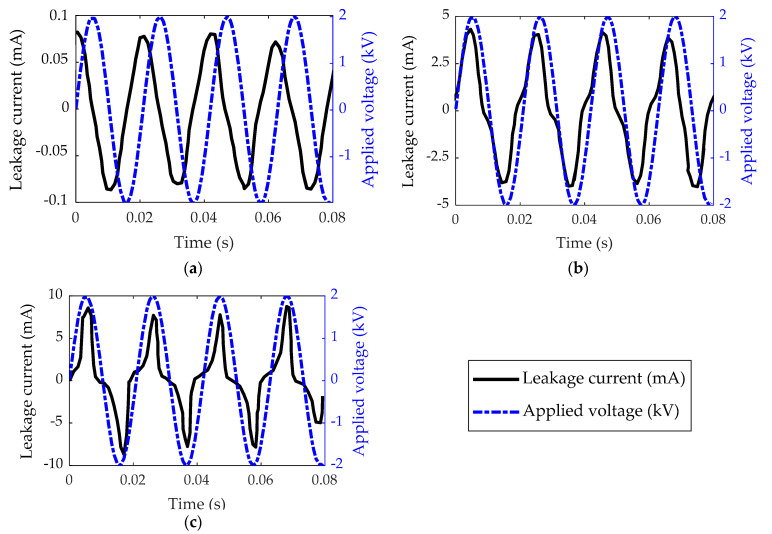
Waveforms of applied voltage and leakage current for insulator type II under different conditions: (**a**) Clean; (**b**) Polluted with fog wetting; (**c**) Polluted with rain wetting rate of 3 L/h.

Under wet pollution tests, the leakage current and applied voltages for low voltage were also investigated. The results of leakage current and applied voltage waveform for insulator type II as an example under clean, pollution with fog, and pollution with high rain is shown in Figure 6. The waveforms were captured at the voltage energization point (1500 V). Figure 6 depicts how the leakage current phase shift angle changes in relation to the applied voltage when the insulator state changes from clean to polluted.

The amount of leakage current is minimal under dry surface conditions, mostly capacitive with a phase shift of 90°. However, in wet conditions, both the phase shift and the amount of the LC alter, as seen in Figure 6. The amplitude of the current increases from 0.078 mA to 4.6 mA and 8.21 mA, respectively. Meanwhile, in high wet and pollution circumstances, the phase difference between applied voltage and current was found to be zero, indicating resistive current conduction.

### 3.3. Electric Field Dependent Pollution Layer Conductivity

The maximum value of surface conductance is projected to reduce when water evaporates from the contaminated layer owing to the Joule heating impact. According to Equation (7), the amount of water evaporation is directly related to the electric field. As a result, the conductivity value is minimum in the low electric field regions. The electric field quickly reduces when it hits the breakdown threshold. The breakdown voltage was obtained experimentally to be around 10 kV/cm. If the contamination layer exceeds this point, it will be dry, placing a highly resistant zone on the insulator surface.

Nonetheless, in the low voltage layer conductance experiments, this requirement needed to be determined and proven experimentally. The test results of the conductance of the pollution layer have been used to represent the parameters of the contaminated layer in the subsequent modeling work. For an insulator under energization voltage, the effective overall electric field of the insulator can be determined using:

(6) $$E=\frac{V}{L}$$

where *L* is the creepage distance of the insulator. The variation in conductance values recorded at two different periods gives the change in conductance, ∆*G_LC_*, for a conductive contamination layer on the surface of the insulator. ∆*G_LC_* reaches maximum conductance level under wet surface circumstances during a whole wetting process, assuming negligible conductance when the dry band occurs. However, the dry band requires some time to be formed under wet pollution. Therefore, the rate of reduction in pollution layer conductance is expressed by:

(7) $$R_{\Delta G}=\frac{\Delta G_{LC}}{t}$$

**Figure 7:**
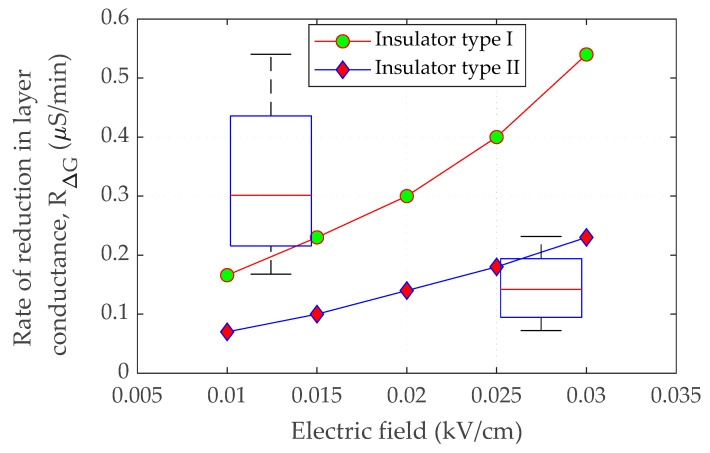
Rate of reduction in conductance for insulator type I and insulator type II as a function of the electric field.

The rate of conductance reduction, *R*_∆*G*_*,* for both insulators as a function of the electric field is shown in Figure 7. The figure shows that with a rise in the electric field, the rate of the growth in the conductance change of the pollution layers is likewise linked to the evaporation rate. The large amplitude of the electric field generates enough heat energy to speed the drying process, resulting in a faster drop rate in surface conductance. For comparison, the box plot of the conductance reduction rate *R*_∆G_ for both insulators is presented in Figure 7. It can be noted that the range of *R*_∆G_ for insulator type I is greater than the *R*_∆G_ range for insulator type II. This situation suggests that the insulator with the high distance between the sheds has a high *R*_∆G_ range. The large amplitude of the electric field generates enough heat energy to speed up the drying process, resulting in a faster drop rate in surface conductance. The relationship between E-field and insulator surface conductance was used for modeling and simulation. Surface conductivity is shown to have an inverse relationship with an electric field. When the contaminated insulator is moist, surface conductance is assumed to be greatest at 8.22 μS for insulator type I and 8.57 μS for insulator type II. When an electric field is increased, the pollution layer conductance is projected to drop by *R*_∆G_ owing to evaporation. However, due to a lack of test data at high energization voltage, the correlation at a high value of electric field could not be inferred. When the sample is energized with a voltage of more than 2.5 kV, violent electrical discharges occur, affecting the experimental data displayed on the oscilloscope. As a result, the plot extrapolation approach predicts features over a wider range of electric fields.

**Figure 8:**
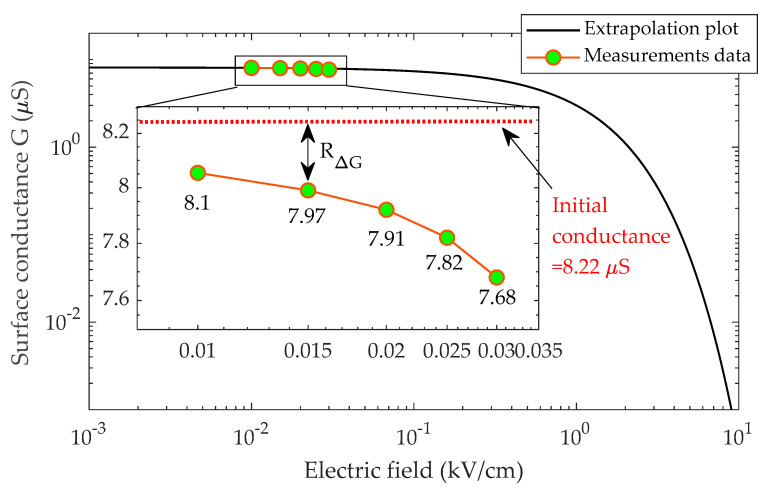
Relationship between the conductance and electric field for insulator type I.

**Figure 9:**
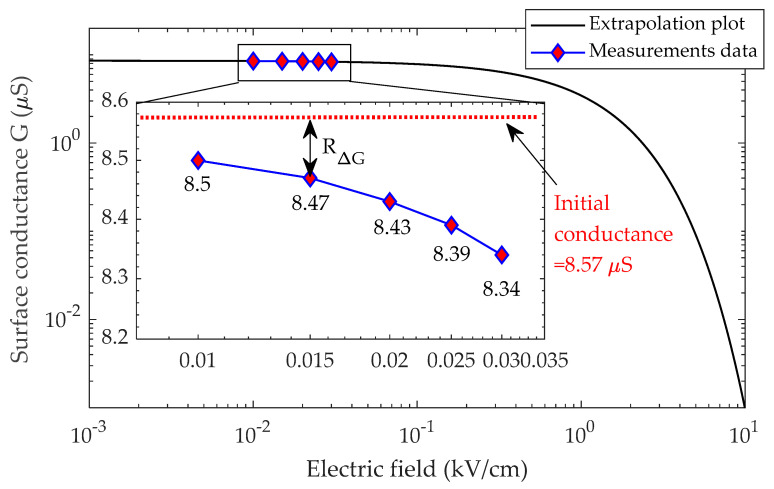
Relationship between the conductance and electric field for insulator type II.

The log-log plot of insulators I and II experimental data, shown in Figure 8 and Figure 9, depicts the relationship between the pollution layer conductance and the electric field. However, due to a lack of empirical data at higher voltage levels, electric fields greater than 0.03 kV/cm could not be calculated. In this work, the breakdown thresholds were found to be 9.8 kV/cm for insulator type I and 8.7 kV/cm for insulator type II when the pollution layer conductance is minimal (0.001 µS). This mean that in the higher-electric field range (10 kV/cm), the surface conductivity is 0.001 S/m, indicating that the drying process is complete.

It is noteworthy that, to produce Figure 8 and Figure 9, a curve-fitting technique was used to determine the electric field and conductance relationship through extrapolation. In the log-log plot, the trend displays an exponential decay, and this relationship is approximated as:

(8) $$G=xe^{-yE}$$

where *G* is pollution layer conductance in µS, *E* is electric field in kV/cm, and *x* and *y* are constants estimated using the curve-fitting technique.

**Figure 10:**
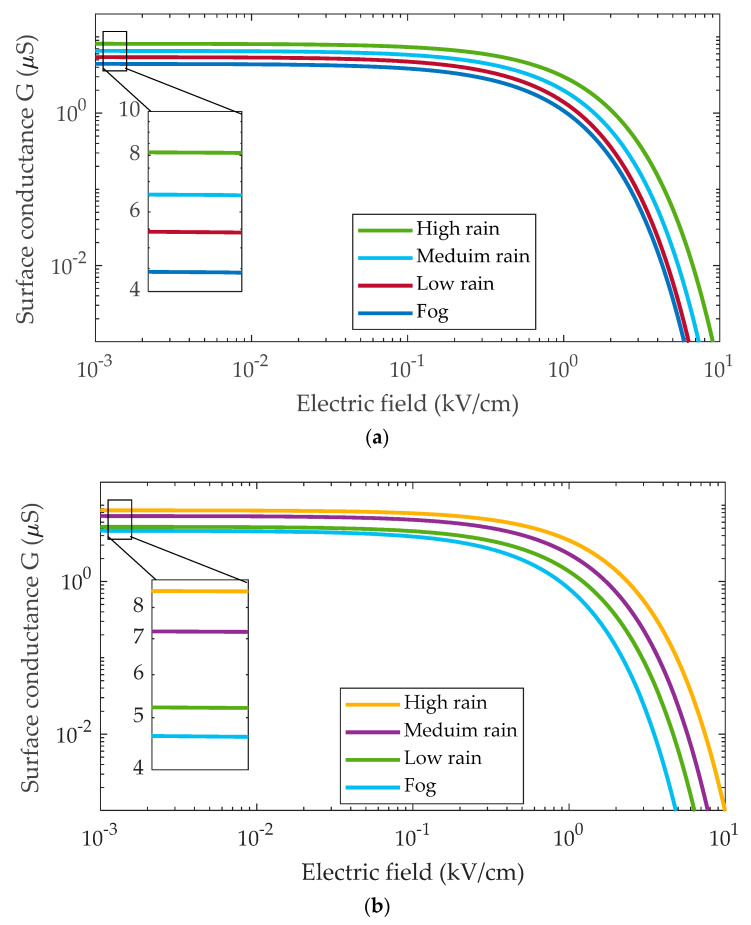
Relationship between the surface conductance and electric field under different wetting levels during a period of testing for (**a**) insulator type I; (**b**) insulator type II.

Figure 10 illustrates the surface conductivity graphs of contaminated layers under fog and rain conditions for both type I and type II insulators. The rain flow rate to the insulator surface is classified based on three levels of rain: high rain, medium rain, and low rain. As can be seen, the curves follow a similar overall pattern, with minor variations in initial conductivity and field threshold. In the insulator I curve, for example, high rain has the greatest surface conductivity of 8.22 µS/m because it is highly saturated with water. On the other hand, the medium and low curves have somewhat small values of conductivity of 6.54 μS/m and 5.36 μS/m, respectively, due to the medium and low rain flow rate. Furthermore, the surface conductivity of insulators under Fog was determined to be 4.32 μS/m for insulator I and 4.85 μS/m for insulator II. The threshold is the field value at which the conductivity of the contaminated layer quickly declines. Places with low wetting have lower field threshold values, implying that dry band development is more likely in these areas. As a result, the pollution model for fog offered indicates the lowest field threshold value, followed by low rain and medium rain with a moderate value. Finally, the high rain flow is confronted with the highest field threshold value.

Referring to Equation (8), the value of the parameter *x* is related to the conductivity values on the surfaces of insulators. In contrast, the parameter *g* is related to the electric field threshold values. It can be observed that when the conductance value increases, so do the *x* value. In contrast, when the electric field threshold value increases, the *y* value decreases. Table 3 lists the values of *x* and *y* for the tested insulators under different conditions.

## 4. Modeling of the Polymeric Outdoor Insulators

**Figure 11:**
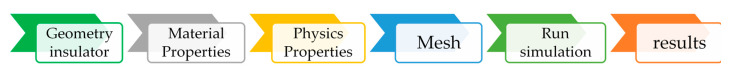
FEM modeling process in COMSOL Multiphysics.

COMSOL Multiphysics software [33] was used to model 33-kV polymeric insulators with and without a contamination layer using the finite element model (FEM). The effect of conductivity on the electric field and current density distribution has been considered in the model. In addition, the quasi-static electric field was employed for numerical analysis [34]. The modeling procedures involve designing the insulator’s geometry, specifying the characteristics of the materials in the insulator sections, implementing the boundary conditions (electrical potentials), providing 33-kV for the HV end and ground (zero voltage) for the other end, and applying the mesh for the model. The modeling process using FEM is shown in Figure 11. The FEM approach is divided into a series of steps, beginning with design and progressing to the application of material properties, followed by the application of physics properties. The mesh is applied to the design prior to performing the simulation. The findings of the simulation may be extracted once the simulation is completed.

### 4.1. Properties of Insulator Material

**Figure 12:**
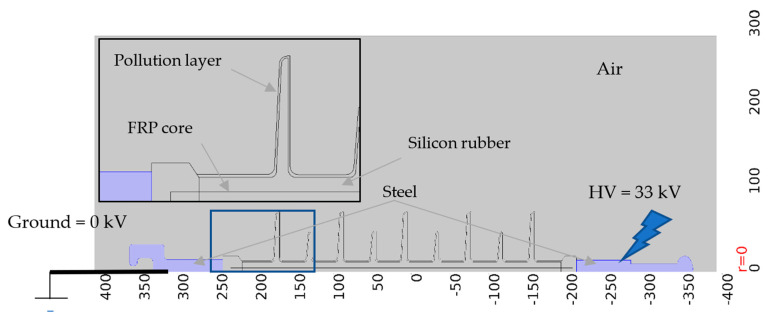
Modeled polymeric insulator structure.

Relative permittivity and conductivity are the main electrical features of the insulator materials examined in this work for simulating the electric field and current density. The insulator structure is shown in Figure 12 and the material properties of the insulator are summarized in Table 4. The polymeric insulator, as illustrated in Figure 12, is made up of a steel fitting on both ends and a fibre reinforced plastic (FRP) core as a load bearing structure, with silicone rubber being utilized as sheds due to its hydrophobic characteristics.

In this study, the permittivity and conductivity values of the air, silicon rubber, FRP core, and steel listed in Table 4 were used. The pollution layer conductivity is defined as a function of an electric field, *p* = *f*(*E*s), which is obtained from the fit in Section 3. When the pollution is in its conductive form the relative permittivity is set at 81, water is the major substance for conductance. Contamination deposition on transmission line insulators is often uneven and heavily influenced by the type and location of the insulator. The pollution in this study is assumed to be homogeneous at a thickness of 0.5 mm throughout the insulator surface to minimize modeling difficulty.

### 4.2. Electrical Characteristics Calculation Using FEM

**Figure 13:**
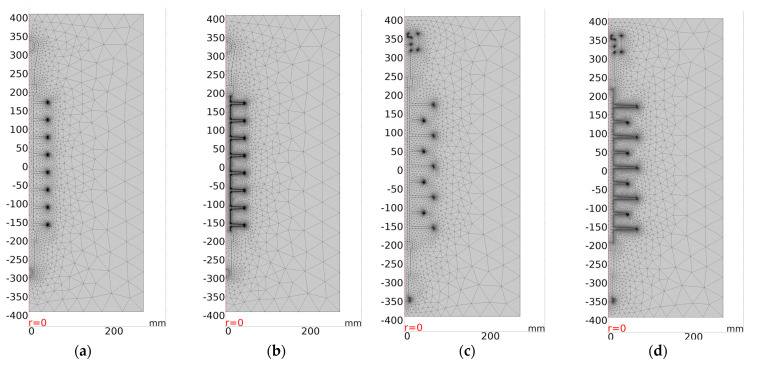
FEM meshing for (**a**) clean type I insulator; (**b**) polluted insulator type I; (**c**) clean insulator type II insulator; (**d**) polluted insulator type II.

To simulate the insulator, an axisymmetric model was used, taking into account the axial symmetry of the insulator. Before calculating the electrical characteristics, the insulators models have meshed. A normal element size with a triangular shape was used in the meshing procedure. Figure 13 demonstrates type I and type II polymeric insulators after meshing under clean and polluted conditions. The mesh properties and a number of degrees of freedom (DOF) solved for each insulator under clean and pollution conditions were listed in table Appendix A (Table A1). A noteworthy aspect of meshing is that the elements were small in size in sharp or curvature areas. The simulation was conducted after meshing, and the insulators’ electrical characteristics, namely potential V, electric field E, and current density J, were obtained. We solved the Laplace equation in the geometry of Figure 12. On each of the boundaries between two materials, we used the electrostatic boundary condition that the normal component of the displacement field is continuous, using the dielectric properties from Table 4.

During the simulation procedure, the following basic equations were utilized in COMSOL software to calculate the electric field:

(9) $$\vec{E}=-\vec{\nabla }V$$

where *E* and *V* represent the electric field and electric potential, respectively. The leakage current density *J* was then determined as a function of conductivity as [36]:

(10) $$\vec{J}=\sigma \vec{E}$$

where *σ* is the pollution layer conductivity. The current density varies with insulator shape, with the greatest current density occurring at shank sections where the circular surface is mainly minor. Increased power dissipation is caused by a rise in electric field and current density that becomes the source of warming and dry band formation.

## 5. Simulation Results and Discussion

### 5.1. Electric Field Distribution

**Figure 14:**
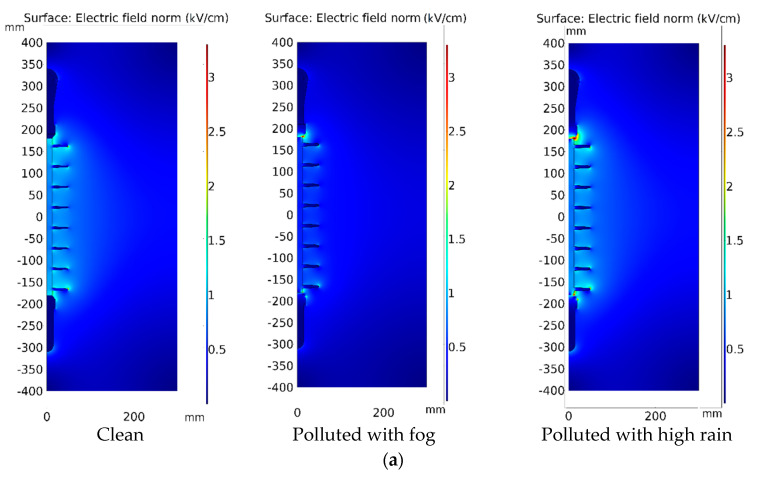

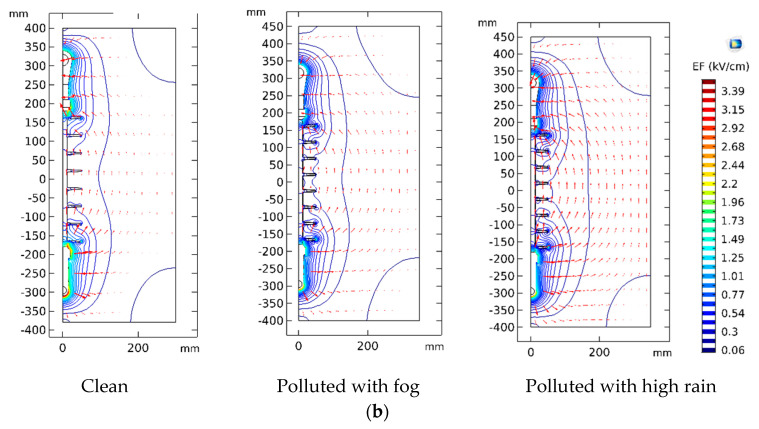
Electric field distribution of insulator type I: (**a**) Surface plot; (**b**) Contour plot.

**Figure 15:**
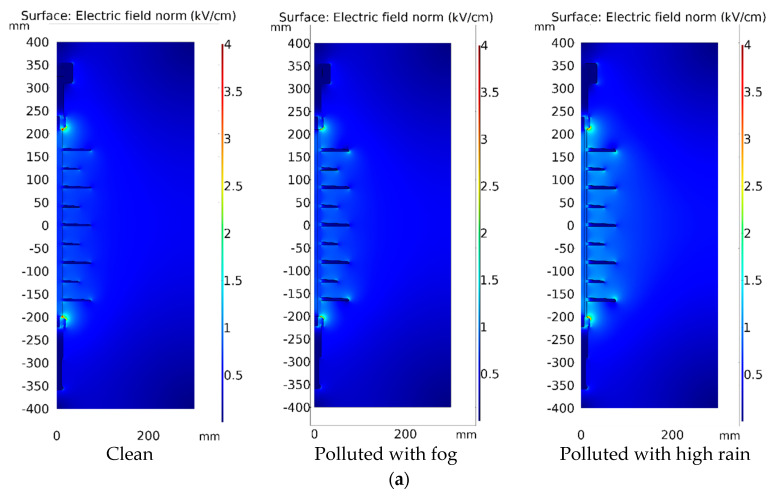

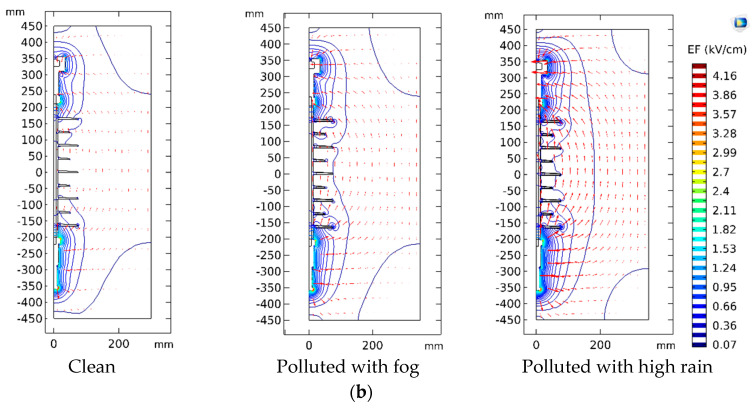
Electric field distribution of insulator type II: (**a**) Surface plot; (**b**) Contour plot.

The distribution of the electric field along the surface of the polymeric insulators was investigated and calculated. Wet contaminated surfaces were simulated using experimentally observed conductivity. The electric field, current density, and pollution layer conductance are the most common modeling parameters observed in the majority of published research [37]. However, the influences of the wetting and drying processes are not taken into consideration in most of these studies. The results of the simulation were used as a control and a comparison. Figure 14 and Figure 15 depict the modeling results of insulators for the electric field distribution, contour, and electric field arrows of the surface under clear, fog, and high rain conditions. As shown in Figure 14 and Figure 15, the electric field weakens in polluted parts under fog circumstances compared to non-polluted regions. If the wetting (rain) increases, the variance in conductivity induces interfacial polarization. It drives charge building at the pollution border, leading to an electric field with a high value on both sides of the pollution region. According to Figure 14b and Figure 15b, it can be seen that the electric field arrow comes out of the positive electrode (which is positively charged) and moves towards the negative electrode (negatively charged). In addition, under heavy rain circumstances, increasing the intensity arrows of the electric field indicates a rise in charge flow, which ultimately raises the field’s strength. It is noteworthy that there is no appreciable variation of the numerical results if the closed geometries of the models are made larger than the ones used in the current simulation.

**Figure 16:**
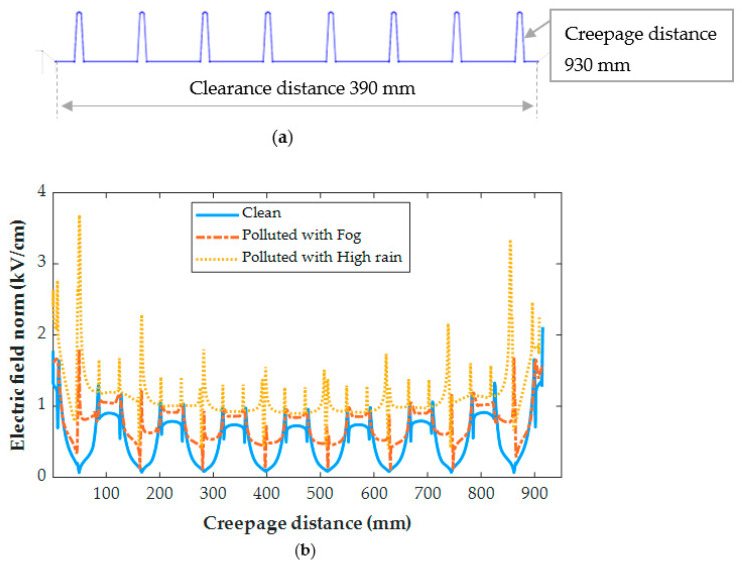
(**a**) Electric field path for insulator type I; (**b**) Electric field distribution for insulator type I under clean, polluted with fog, and polluted high rain conditions.

**Figure 17:**
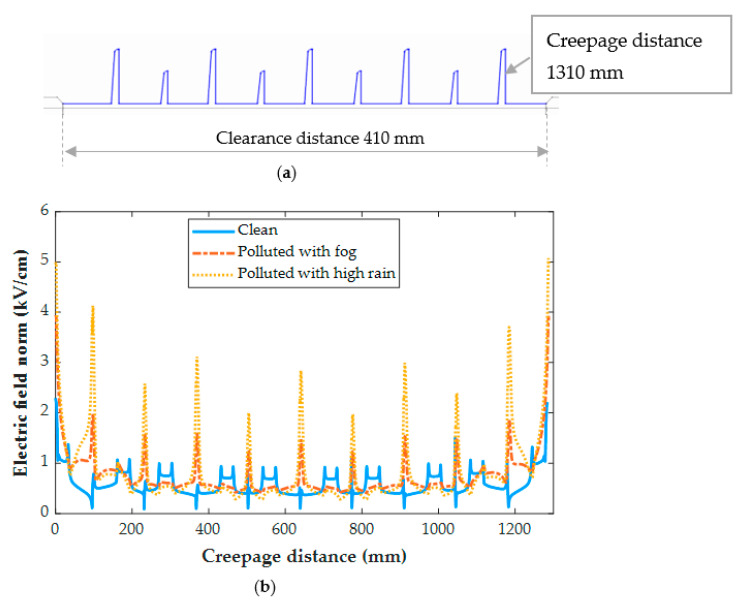
(**a**) Electric field path for insulator type I; (**b**) Electric field distribution for insulator type II under clean, polluted with fog, and polluted with high rain conditions.

**Figure 18:**
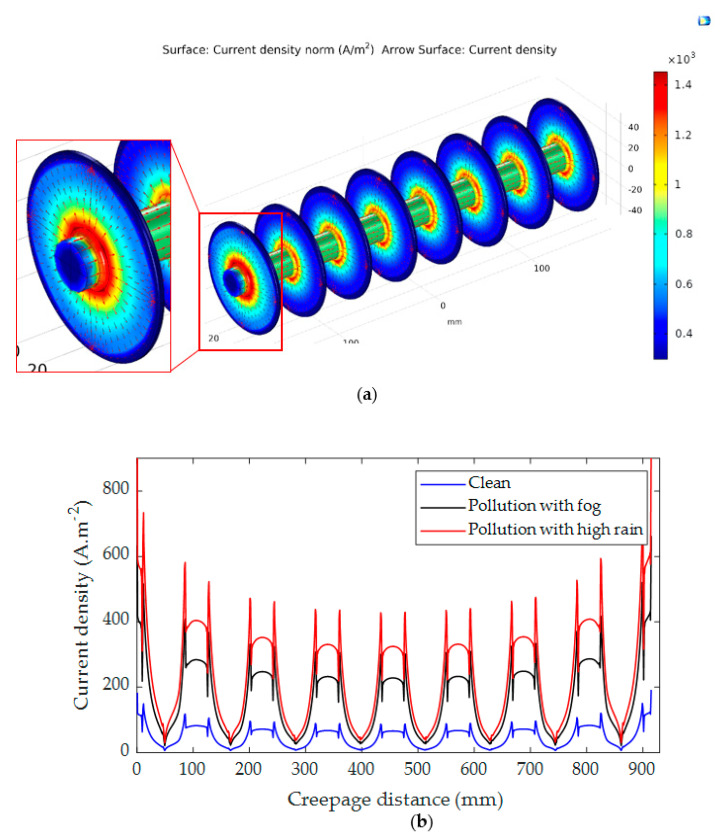
Current density distribution for insulator type I: (**a**) 3-D simulation view; (**b**) Current density distribution along the insulator.

**Figure 19:**
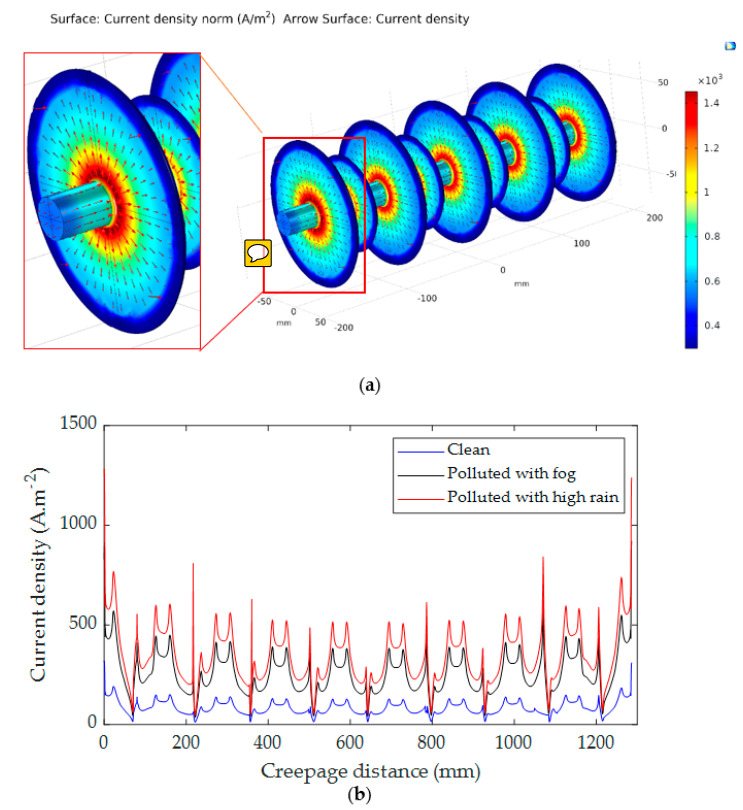
Current density distribution for insulator type II: (**a**) 3-D simulation view; (**b**) Current density distribution along the insulator.

Figure 16 and Figure 17 demonstrate the electric field distribution under fog and rain in comparison to the clean state for insulator type I and insulator type II, respectively. The contamination models, as shown in Figure 18 and Figure 19, are distinguished by nonlinear field-dependent conductivity. There is a little variance between the field profiles. The insulators under the Fog and rain face the increase in an electric field. When insulator type I was exposed to wetness, the electric field increased along the insulator. It is worth noting that the electric field has been considerably increased in the shed’s region, particularly near terminals. When the insulator was exposed to fog and rain, the electric field rose from 0.3 kV/cm to 1.74 kV/cm and 3.68 kV/cm, respectively. In insulator II, the terminals are subjected to field stress increases ranging from 2.2 kV/cm to 4.5 kV/cm, resulting in a 104% raising in the field. In shank areas, there is also an increase in the electric field of roughly 23%. Referring to Figure 14 the insulator surface conductivity is high in the high rain condition. The rise in the electric field, particularly under heavy rain, is caused by an increase in surface conductivity [38]. This explains the obtained high value of the electric field under high rain conditions in Figure 16 and Figure 17.

These variations are caused by a decrease in surface conductivity upon exceeding the drying threshold in higher field locations. In this simulation, the pollution model produces a field distribution with a succession of peaks at various positions on the polymeric surface. It has been discovered that the field expands also on the sheds and most wetted surfaces. Similarly, a considerable field increase is seen in the shed regions, suggesting a sensitive location to electric discharge operations. On the other hand, the shed surfaces, which are characterized by significant wetness and include the one nearest to the HV terminal, indicate a beneficial shift in the electric field. This might be owing to the areas that are subjected to significant wetting activity. Dry bands and electrical discharges are widely detected on the shank areas in most experimental investigations, and they match well with the modeling results of this study. With the increase of the pollution layer conductivity, the increase in the electric field intensity is more noticeable.

In comparison between the electric field (EF) results of tested insulators, the EF on the insulator type II is higher than on type I due to the larger diameter of the extremely large shed on the insulator type II, which resulted in the formation of the dry bands with large areas. In addition, the insulator type II’s larger leakage distance has an influence on the EF increase.

### 5.2. Current Dencity Distribution

**Figure 20:**
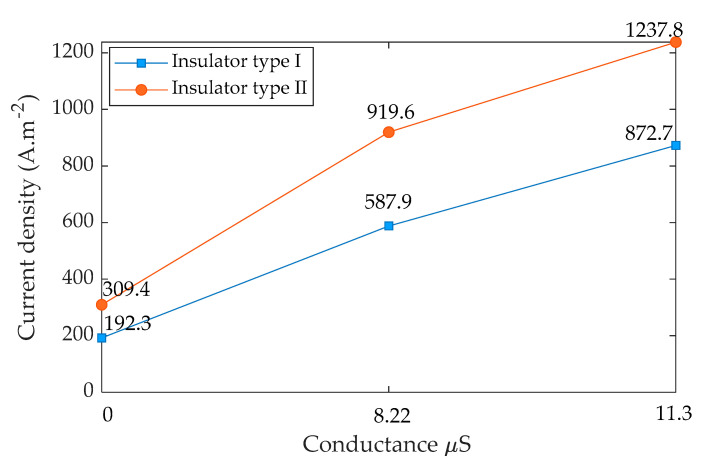
Relationship between current density and conductance of insulator type I and insulator type II.

The distribution of current density on the insulator surface with different electrical conductivities is critical. Therefore, the current density distribution and its arrow direction along insulators’ surfaces were simulated. The simulation was carried out in 3D geometrical representations as shown in Figure 18a and Figure 19a. It can be seen that the high current density areas are mostly located in the cross of the sheds and the insulator’s shanks and around the electrodes because the high current density is often formed in those areas where the insulator diameter is the smallest. Figure 18b and Figure 19b show the distribution of current density along the insulator under the clean and contamination with fog and rain. The results indicate that the current density has a maximum value on the polluted insulator with rain wetting for both insulators. The pollution layer conductance values were 8.22 µS and 11.23 µS, respectively, when the insulator was exposed to fog and heavy rain as mentioned in Section 4.1. The relationship between the pollution layer conductance and current density for both insulators is presented in Figure 20. As shown in Figure 20, by increasing the conductance of the pollution layer, the current density increases significantly. For high conductance, the increasing rate of the current density becomes minor.

**Figure 21:**
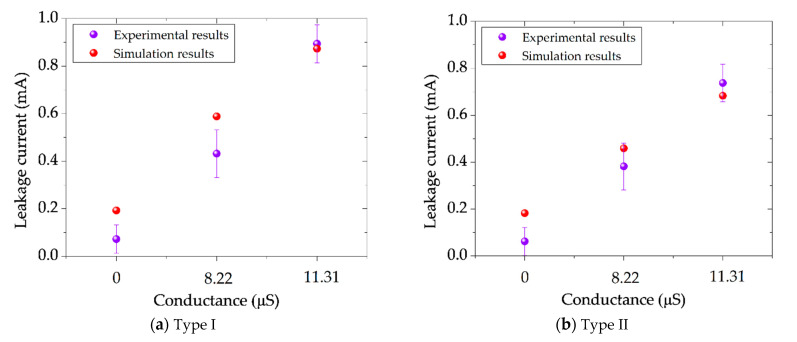
(**a**) Comparison between simulation and experimental results of the leakage current under three different conductance conditions for insulator type I; (**b**) Comparison between simulation and experimental results of the leakage current under three different conductance conditions insulator type II.

To determine the leakage current using the FEM model, the current density was integrated over the surface of the insulator. Then, the leakage current results obtained from the FEM model were compared with the experimental findings. Figure 21 depicts the relationship between the leakage current of the insulator and the contamination layer conductance obtained from experiments in Figure 6 and simulation. As can be observed, there is an error in the leakage current simulation on the insulator’s surface compared to experimental results. However, the error is still reasonable and reflects the FEM model’s strong performance in simulating the leakage current.

## 6. Conclusions

The development of a nonlinear pollution model has been performed to estimate the electric field distribution along the insulators. A contaminated layer conductance test was carried out on two insulators with different profiles to generate the relationship between the surface conductance and electric field. Under uniform wetting, the observed breakdown voltage threshold for both insulators was not greater than 10 kV/cm. The extrapolation plot of the surface conductance curve was used to extract the electrical characteristics of the pollutant to be utilized in FEM modeling. The wetting and drying are considered and characterized using simplified assumptions to define pollution under fog and three flow rates of rain conditions. The proposed model indicated a distribution of both electric field and current density with a sequence of peaks on the surface of the polymeric insulators. Due to applied rain wetting on the insulators, the terminals experienced local stress increases ranging from 0.3 kV/cm to 3.6 kV/cm for insulator type I and 2.2 kV/cm to 4.5 kV/cm for insulator type II. Minor increases in the electric field were also detected in the shanks of the insulators, owing mostly to a decrease in electric conductivity on the surface upon exceeding the drying threshold at the regions with the higher fields. Similarly, the current density has the maximum value around the electrodes and the cross of sheds with shanks. The findings of the experiment and simulation revealed that the insulator profile had an influence on the electrical properties.

## Figures and Tables

**Table 1:** Dimensions of type I and type II insulators.

Parameter in (mm)	*D*	*d*	*S*	*H*	*L*	*L/H*	No. of Sheds
Type I	98	-	45	390	930	2.96	8
Type II	130	90	41	410	1310	2.1	9

**Table 2:** Maximum values of conductance for five tests, average conductance, and standard deviation of surface conductance when the insulators were saturated with water.

Test No.	Conductance						Standard Deviation
	1	2	3	4	5	Average	
Type I (μS)	8.758	8.477	8.661	8.637	8.358	8.59	0.15
Type II (μS)	8.25	8.154	8.198	8.26	8.283	8.22	0.052

**Table 3:** The values of the fitting function parameters *x* and *y* under different wetting conditions.

Insulator	Wetting	*x*	*y*	Threshold Field
Type I	Fog	4.58	1.234	4.76
	Low rain	5.87	1.063	4.98
	Medium rain	6.92	0.953	6.42
	High rain	8.43	0.914	8.71
Type II	Fog	4.92	1.185	3.93
	Low rain	6.21	0.975	5.42
	Medium rain	7.71	0.938	6.83
	High rain	9.24	0.884	9.86

**Table 4:** Material properties of insulator model.

Materials	Relative Electrical Permittivity, *ε*_r_	Conductivity, *σ* (S/m)
Air	1	10^−13^
Silicone rubber	4.3	10^−12^
FRP core	7.2	10^−14^
Steel [35]	1	11 × 10^5^

## Appendix A

**Table A1:** Simulation parameters of insulators mesh.

**Insulator Type**	**Elements Types**			**Minimum Elements** **Quality**	**DOF**
	**Triangle**	**Edge**	**Vertex**		
Type I clean	16,740	905	134	0.4662	33,551
Type I polluted	36,424	3190	236	0.4068	72,903
Type II clean	22,235	1290	166	0.4902	76,284
Type II polluted	43,853	4202	260	0.3608	87,774
